# Partial nephrectomy provides equivalent oncologic outcomes and better renal function preservation than radical nephrectomy for pathological T3a renal cell carcinoma: A meta-analysis

**DOI:** 10.1590/S1677-5538.IBJU.2020.0167

**Published:** 2020-11-18

**Authors:** Huan Deng, Yan Fan, Feifei Yuan, Li Wang, Zhengdong Hong, Jinfeng Zhan, Wenxiong Zhang

**Affiliations:** 1 The Second Affiliated Hospital of Nanchang University Department of Thoracic Surgery Nanchang China Department of Thoracic Surgery, The Second Affiliated Hospital of Nanchang University, Nanchang, China; 2 The Second Affiliated Hospital of Nanchang University Department of Urology Nanchang China Department of Urology, The Second Affiliated Hospital of Nanchang University, Nanchang, China; 3 Nanchang University Jiangxi Medical College Nanchang China Jiangxi Medical College, Nanchang University, Nanchang, China; 4 The Second Affiliated Hospital of Nanchang University Department of Nephrology Nanchang China Department of Nephrology, The Second Affiliated Hospital of Nanchang University, Nanchang, China

**Keywords:** Nephrectomy, Carcinoma, Renal Cell, Meta-Analysis [Publication Type]

## Abstract

**Purpose:**

Radical nephrectomy (RN) is the standard surgical type for pathological stage T3a (pT3a) renal cell carcinoma (RCC). Recently, some studies have suggested equivalence between partial nephrectomy (PN) and RN for oncologic control and have shown the benefits of PN for better renal function. We conducted this meta-analysis to assess oncologic outcomes, perioperative outcomes and renal function between two groups among patients with pT3a RCC.

**Materials and methods:**

PubMed, Scopus, Web of Science, Science Direct, Ovid MEDLINE, The Cochrane Library, Embase and Google Scholar were searched for eligible articles. The endpoints of the final analysis included overall survival (OS), cancer-specific survival (CSS), recurrence-free survival (RFS), surgical complications, operative time, estimated blood loss (EBL), serum creatinine and estimated glomerular filtration rate (eGFR).

**Results:**

Twelve studies of moderate to high quality, including 14.152 patients, were examined. PN showed superiority for renal functional preservation, providing higher eGFR (WMD=12.48mL/min; 95%CI: 10.28 to 14.67; P <0.00001) and lower serum creatinine (WMD=-0.31mg/dL; 95%CI: −0.40 to −0.21; P <0.00001). There were no significant differences between PN and RN regarding operative time, EBL, surgical complications, OS, RFS and CSS. Despite inherent selection bias, most pooled estimates were consistent in sensitivity analysis and subgroup analysis. More positive margins were found in the PN group (RR=2.42; 95%CI: 1.25-4.68; P=0.009).

**Conclusions:**

PN may be more suitable for treating pT3a RCC than RN because it provides a similar survival time (OS or RFS) and superior renal function. Nevertheless, this result is still disputed, and more high-quality studies are required.

## INTRODUCTION

Renal cell carcinoma (RCC) is the eighth most common type of cancer in the United States, with an incidence of 65.340, and caused 14.970 deaths in 2018 (1). Local RCC is the most common manifestation, and nearly one-third of patients are diagnosed with T3-T4 RCC (2). Recently, the oncologic outcomes of partial nephrectomy (PN) were found to have oncologic results similar to those of radical nephrectomy (RN) (3).

PN is recommended by the European Association of Urology (EAU) and National Comprehensive Cancer Network (NCCN) guidelines as the standard choice for T1a-b RCC (4). Additionally, some articles have shown that PN for T2 or greater renal tumors may offer oncologic outcomes similar to those of RN (5). The most attractive and beneficial feature of PN compared with RN is better renal function (6), which might decrease the risk of cardiovascular and metabolic events that may ultimately translate into better overall survival (OS) (7). However, the only randomized control trial (RCT) EORTC 30904 failed to show significant advantages that favored PN in these terms, despite showing oncologic similarity (3). Therefore, it remains controversial whether PN is a feasible choice for pathological T3a (pT3a) RCC.

To resolve this controversy, this article systematically evaluated and analyzed the therapeutic efficacy of PN and RN among patients with pT3a RCC to evaluate OS, cancer-specific survival (CSS), recurrence-free survival (RFS), surgical complications, perioperative outcomes and renal functions between PN and RN to provide evidence-based data for patients with pT3a RCC with regard to the selection of surgical procedures.

## MATERIALS AND METHODS

Our meta-analysis was performed in accordance with Preferred Reporting Items for Systematic Review and Meta-Analysis (PRISMA) guidelines (Registration information: CRD42020153787).

### Search strategy

PubMed, Scopus, Web of Science, Science Direct, Ovid MEDLINE, The Cochrane Library, Embase and Google Scholar were searched up to April 15, 2019, to identify relevant articles comparing PN to RN for pT3a RCC. The following terms were used: “renal cell carcinoma”, “pathological T3a”, “partial nephrectomy” and “radical nephrectomy”. We also searched the references of included studies to find further eligible studies.

### Inclusion criteria

Studies that satisfied the following criteria were included: 1) patients diagnosed with pT3a RCC; 2) comparison of PN with RN; 3) final outcomes of RFS, OS, CSS, surgical complications, estimated blood loss (EBL), operative time, serum creatinine and estimated glomerular filtration rate (eGFR). We excluded reviews lacking raw data, meta-analyses, conference abstracts, animal experiments and articles with repeated data.

### Data extraction

Two investigators abstracted the following information independently: year of publication, first author, study origin, study period, study design, number of participants, participant characteristics (age, sex, tumor size, pathological type, surgical approach and so on), oncologic outcomes (OS, RFS, CSS), perioperative outcomes (EBL, operative time, positive margins), surgical complications (intraoperative and postoperative complications) and renal function (eGFR, serum creatinine). A third investigator settled differences in all situations.

We used the multivariable adjusted hazard ratio (HR), which takes into consideration the quantity and time of events instead of OR, to analyze oncologic outcomes. HRs and 95%CIs were obtained directly if Cox multivariate survival analysis was conducted; otherwise, HRs and 95%CIs were extracted from Kaplan-Meier curves according to Tierney et al. (8, 9). Some 3-year all-cause mortality, 5-year all-cause mortality, 3-year recurrence rate, 5-year recurrence rate, 2-year cancer-specific mortality (CSS) and 5-year CSS data were also extracted from survival curves because of the lack of available data in the included articles.

### Quality assessment

The quality of each study was assessed using the Newcastle-Ottawa Scale (NOS) for retrospective studies, which includes questions on three major projects: selection, comparability and exposure. A total score of 8-9 points was considered high-quality; 6-7 was considered medium-quality (10).

## Statistical analysis

This meta-analysis was performed using Review Manager (version 5.2, The Nordic Cochrane Centre) and STATA (version 12.0, Stata Corp). Risk ratios (RR) with 95% confidence intervals (CIs) were used to analyze 3-year all-cause mortality, 5-year all-cause mortality, 3-year recurrence rate, 5-year recurrence rate, 2-year CSS, 5-year CSS and positive margins (RR >1 supports PN; RR <1 supports RN). Hazard ratios (HR) with 95%CIs were used to analyze OS, RFS and CSS (HR >1 supports RN; HR <1 supports PN). Weighted mean difference (WMD) and 95%CIs were employed to assess operative time, EBL, eGFR and serum creatinine. Subgroup analysis of HR of OS, RFS and CSS were performed to determine whether the results would vary according to upstaging, adjustment/matching, study center, tumor size and follow-up time. Heterogeneity was examined using the χ^2^ test and I^2^ statistic. If I^2^>50% or P <0.1 for the χ^2^ test, reflecting significant heterogeneity, the random-effects model was used; if not, the fixed-effects model was used. To enhance robustness, sensitivity analysis was performed to determine the effects of variables. Publication bias was evaluated using Begg's test and Egger's test. P <0.05 indicated statistical significance.

## RESULTS

### Search results and study quality assessment

**Figure 1:**
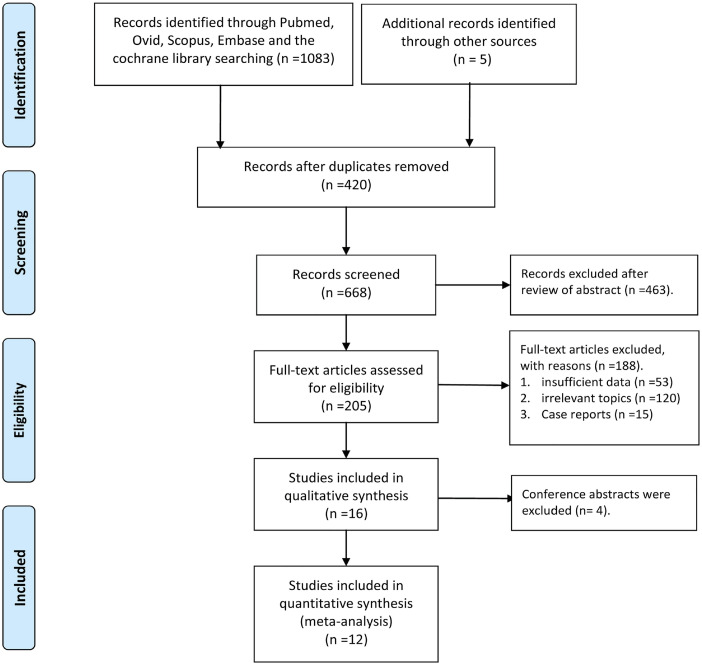
Flow chart of study selection.

Figure-1 shows the process of study selection. Ultimately, 12 studies including 14.152 patients (2486 PN and 11.666 RN) were selected for this meta-analysis (11–22). Of the 12 studies, four were high quality and eight medium quality (Table-S1). Table-1 provides the baseline characteristics and major evaluation indices of the included articles.

**Table S1:** Quality assessment of all included studies according to Newcastle-Ottawa Scale.

Study	Selection				Comparability	Exposure			Quality score
	Is the case definition adequate?	Representativeness of the cases	Selection of Controls	Definition of Controls	Comparability of cases and controls on the basis of the design or analysis	Ascertainment of exposure	Same method of ascertainment for cases and controls	Non-Response rate	
Jeldres, et al. (11)	★	★		★	★★	★	★	★	8
Hansen, et al. (12)	★	★		★	★★	★	★		7
Polo et al. (13)	★	★		★	★	★	★	★	7
Oh et al. (14)	★	★		★	★	★	★	★	7
Jeong et al. (15)	★	★		★	★	★	★	★	7
Nayak et al. (16)	★	★		★	★	★	★	★	7
Shah et al. (17)	★	★		★	★	★	★	★	7
Andrade et al. (18)	★	★		★	★★	★	★	★	8
Peng et al. (19)	★	★		★	★★	★	★	★	8
Shvero et al. (20)	★	★		★	★	★	★	★	7
Srivastava et al. (21)	★	★		★	★★	★	★	★	8
Lee et al. (22)	★	★		★	★	★	★	★	7

**Table 1:** Characteristics of the included studies.

Study		Study period	Study design	Study Origin	Groups	patients (n)	Tumor size(cm)	ccRCC(n)	Fuhrman grade III/IV(n)	Surgical approach	Adjustment/matching	FU (month)	SQ
Jeldres et al, (11)	2009	1984-2001	RTP, MI	Canada, Italy, France	PN vs. RN	30/63	1.5-9.5/1.5-10.5	27/58	12/32	NS	Yes	50.4	8
Hansen et al, (12)	2012	1988-2008	RTP, MI	USA	PN vs. RN	477/477	2.4–4.5/2.5–4.8	354/355	NS	NS	Yes	NS	7
Polo et al, (13)	2012	1994-2009	RTP, NS	France	PN vs. RN	10/33	2.7/6.0	5/25	3/15	NS	No	45	7
Oh et al, (14)^a^	2014	2000-2010	RTP, MI	Korea	PN vs. RN	45/298	3.50 ± 1.55/7.99 ± 3.68	36/247	23/211	Open/Lap/Rob	No	43	7
Jeong et al, (15)	2016	2001-2013	RTP, SC	Korea	PN vs. RN	37/54	NS	NS	58 c	Open/Lap/Rob	No	50.8	7
Nayak et al, (16)	2016	2009-2015	PRO	Canada	PN vs. RN	66/68	3.5-5.7	NS	76 c	Open/MIS	No	23	7
Shah et al, (17)	2017	2006-2014	RTP, SC	USA	PN vs. RN	49/91	4.2/5.5	41/86	NS	Lap/open	No	38	7
Andrade et al, (18)	2017	2005-2015	RTP, SC	USA	PN vs. RN	70/70	3.0–5.2/3.9–5.4	50/64	43/40	Rob	Yes	20	8
Peng et al, (19)	2017	2007-2012	RTP, SC	China	PN vs. RN	18/18	5.27±1.50/5.03±1.42	13/13	6/6	Open/Lap	Yes	35.5	8
Shvero et al, (20)	2018	1987-2015	RTP, MI	Israel	PN vs. RN	48/86	2.8-5.2/5-9.5	41/67	25/53	NS	No	55.2/48.8	7
Srivastava et al, (21) b	2018	1998-2013	RTP, MI	USA	PN vs. RN	1579/10250	2.5-5.0/4.9-9.0	791/5997	541/4482	NS	No	36/37	8
Lee et al, (22)	2018	1997-2016	RTP, SC	Korea	PN vs. RN	57/158	3.7–6.2	175 c	145 c	LAP	No	39	7

**RTP** = retrospective; **PRO** = prospective; **MI** = multi-institutional; **SC** = single center; **FU** = Follow-up; **Lap** = laparoscopic; **Rob** = robotic; ccRCC = clear-cell renal cell carcinoma; **MIS** = minimally invasive surgery; **NS** = not specified; **SQ** = study quality according to the Newcastle-Ottawa scale

a = The group reported two separate subgroup analyses for the same data set.

b = The group reported three separate subgroup analyses for the same data set.

c = These studies only provide overall numbers, without providing numbers of PN and RN groups respectively.

### Oncologic outcomes

We assessed oncologic outcomes between PN and RN groups based on OS, RFS, and CSS.

**Figure 2:**
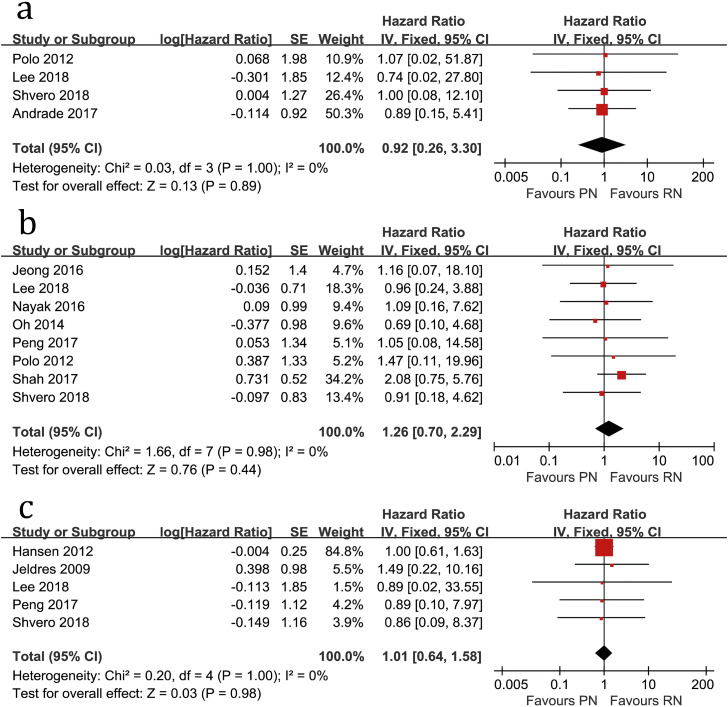
Forest plots of HR of OS (a), RFS (b) and CSS (c) associated with PN versus RN.

Four studies compared the HR of OS (heterogeneity: P=1.00, I^2^=0%). No significant difference was found between PN and RN (HR=0.92, 95%CI: 0.26-3.30, P=0.89; Figure-2A).

Eight studies compared the HR of RFS (heterogeneity: P=0.98, I^2^=0%). No significant difference was found (HR=1.26, 95%CI: 0.70-2.29, P=0.44; Figure-2B).

Five studies compared the HR of CSS (heterogeneity: P=1.00, I^2^=0%). No significant difference was found (HR=1.01, 95%CI: 0.64-1.58, P=0.98; Figure-2C).

**Figure 3:**
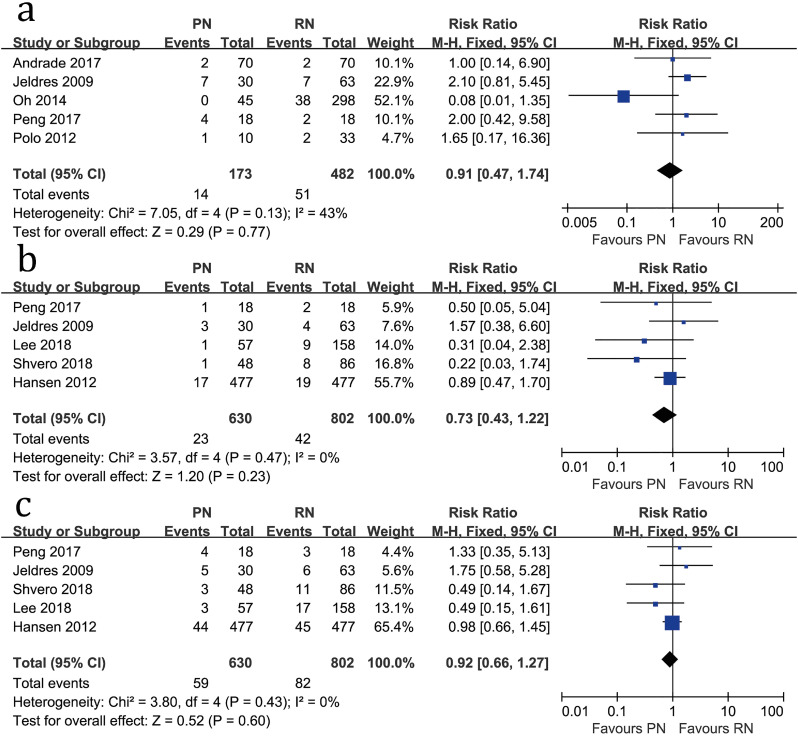
Forest plots of total CSS (a), 2-year-CSS (b) and 5-year CSS(c) associated with PN versus RN.

Five studies compared total CSS (heterogeneity: P=0.13, I^2^=43%). No significant difference was found between PN and RN (RR=0.91, 95%CI: 0.47-1.74, P=0.77; Figure-3A). Moreover, there were no significant differences regarding 2-year CSS (RR=0.73, 95%CI: 0.43-1.22, P=0.23; Figure-3B) and 5-year CSS (RR=0.92, 95%CI: 0.66-1.27, P=0.60; Figure-3C).

**Figure 4:**
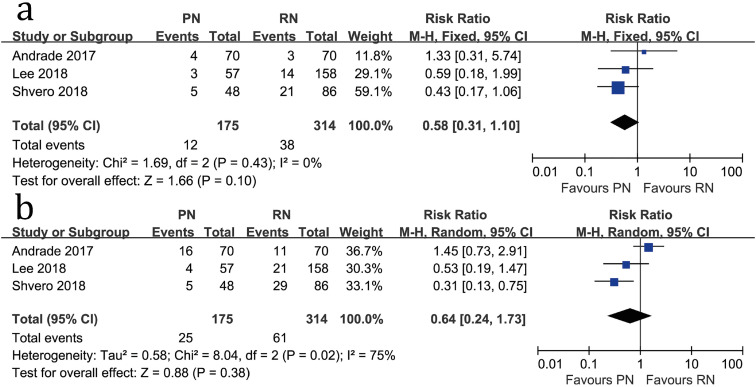
Forest plots of 3-year all-cause mortality (a) and 5-year all-cause mortality (b) associated with PN versus RN.

**Figure 5:**
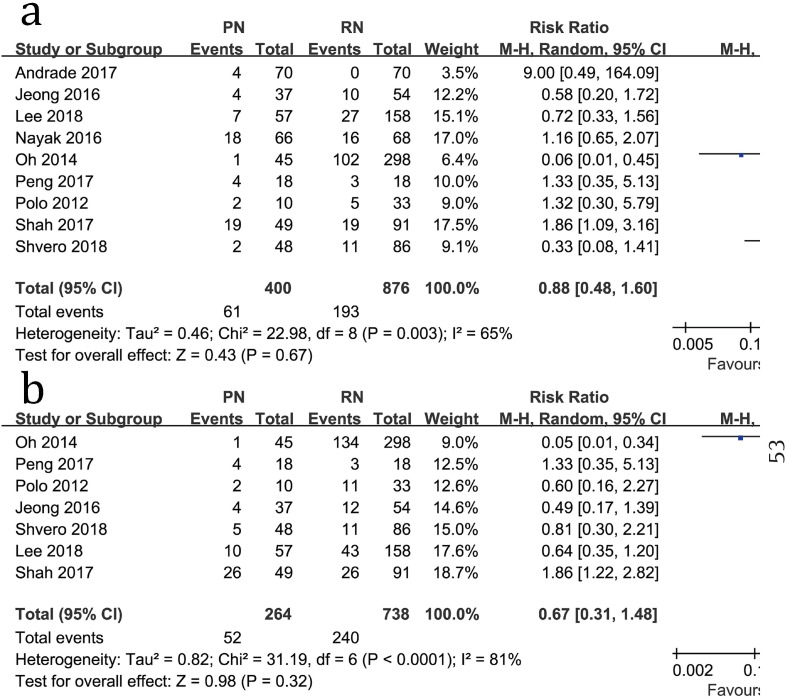
Forest plots of 3-year recurrence rate (a) and 5-year recurrence rate (b) associated with PN versus RN.

There was no significant difference between the two groups regarding 3-year all-cause mortality (RR=0.58, 95%CI: 0.31-1.10, P=0.10; Figure-4A) or 5-year all-cause mortality (RR=0.64, 95%CI: 0.24-1.73, P=0.38; Figure-4B).

Furthermore, no significant difference was found between the two groups for the 3-year recurrence rate (RR=0.88, 95%CI: 0.48-1.60, P=0.67; Figure-5A) or the 5-year recurrence rate (RR=0.67, 95%CI: 0.31-1.48, P=0.32; Figure-5B).

### Surgical complications

Only one included study (Oh, 2014) reported intraoperative and postoperative complications, with no significant differences regarding intraoperative complications (15.6% vs. 14.4%, P=0.842) or postoperative complications (13.3% vs. 12.4%, P=0.844).

Furthermore, no significant differences were found between the two groups regarding prolonged bleeding (2.2% vs. 4.4%, P=0.499); wound problems (2.2% vs. 1.7%, P=0.795); urine leakage (0% vs. 0.3%, P=0.697); prolonged ileus (2.2% vs. 2.7%, P=0.856) and others (6.7% vs. 3.4%, P=0.278) (14).

### Perioperative outcomes

**Figure 6:**
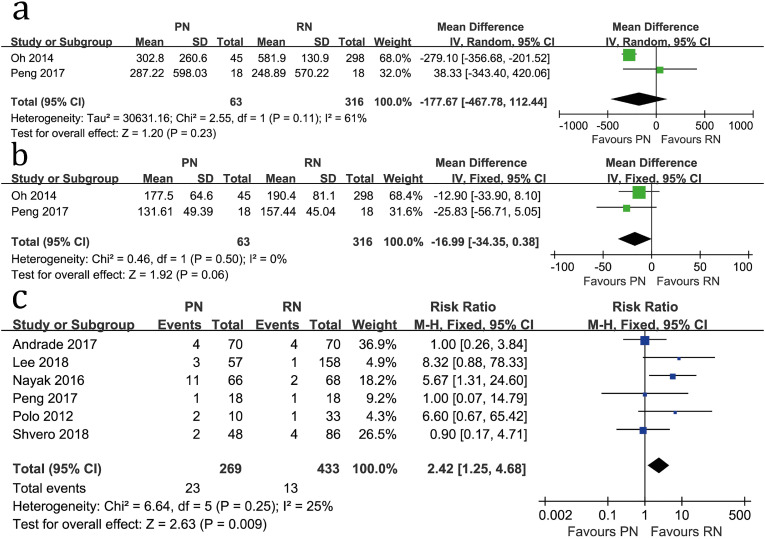
Forest plots of EBL (a), operative time (b) and positive margins (c) associated with PN versus RN.

Two studies compared EBL (heterogeneity: P=0.11, I^2^=61%). No significant difference was found (WMD= −177.67mL; 95%CI: −467.78mL to 112.44mL; P=0.23; Figure-6A).

Two studies compared operative time (heterogeneity: P=0.50, I^2^=0%). No significant difference was found (WMD= −16.99 min; 95%CI: −34.35 min to 0.38 min; P=0.06; Figure-6B).

Six studies compared positive margins (heterogeneity: P=0.25 I^2^=25%), and PN exhibited a higher incidence (RR=2.42; 95%CI: 1.25-4.68; P=0.009; Figure-6C).

### Postoperative renal function

**Figure 7:**
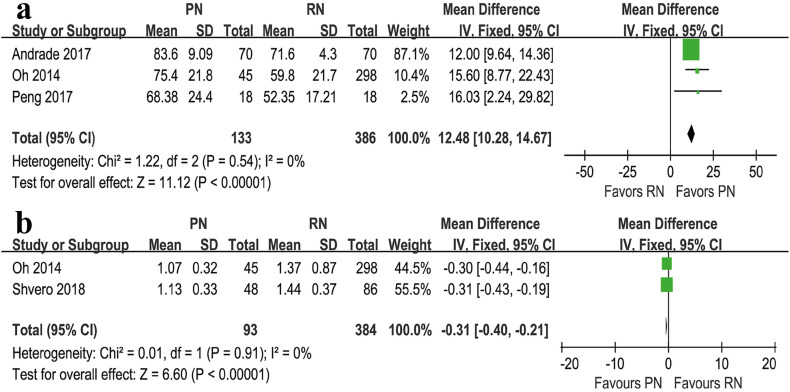
Forest plots of eGFR (a) and serum creatinine (b) associated with PN versus RN.

Three studies compared eGFR (heterogeneity: P=0.54, I2=0%). PN had a higher eGFR compared with RN (WMD=12.48mL/min; 95%CI: 10.28mL/min to 14.67mL/min; P <0.00001; Figure-7A).

Two studies compared serum creatinine (heterogeneity: P=0.91, I^2^=0%), with RN being associated with higher levels compared with PN (WMD= −0.31mg/dL; 95%CI: −0.40mg/dL to −0.21mg/dL; P <0.00001; Figure-7B).

### Subgroup analysis

To determine whether the oncologic outcomes of PN versus RN were robust across subgroups, pooled HRs of OS, RFS and CSS were estimated by upstaging, adjustment/matching, study center, tumor size and follow-up time. No statistically significant differences were found in any of the subgroup analyses of HR of OS, RFS and CSS between PN and RN (Table-2).

**Table 2:** Subgroup analyses for overall survival, recurrence free survival and cancer specific survival.

Group		OS				RFS				CSS			
		No.of studies	HR (95% CI)	P	I2 (%)	No.of studies	HR (95% CI)	P	I2 (%)	No.of studies	HR (95% CI)	P	I2 (%)
**Total**		**4**	**0.92(0.26-3.30)**	**0.89**	**0**	**8**	**1.26(0.70-2.29)**	**0.44**	**0**	**5**	**1.01(0.64-1.58)**	**0.98**	**0**
**Upstaging**												
	Yes	1	0.74(0.02-27.80)	0.87	NA	4	1.47(0.71-3.06)	0.30	0	1	0.89(0.02-33.55)	0.95	NA
	No	3	0.95(0.24-3.71)	0.94	0	4	0.91(0.32-2.55)	0.86	0	4	1.00(0.64-1.58)	0.99	0
**Adjustment/matching**													
	Yes	1	0.89 (0.15, 5.41)	0.9	NA	1	1.05 (0.08, 14.58)	0.94	NA	3	1.01 (0.64, 1.61)	0.95	0
	No	3	0.94 (0.15, 5.79)	0.95	0	7	1.27 (0.69, 2.35)	0.44	0	2	0.87 (0.13, 5.97)	0.89	0
**Study center**													
	Single	2	0.86(0.17-4.32)	0.85	0	4	1.49(0.70-3.16)	0.30	0	2	0.89(0.14-5.82)	0.9	0
	Multiple	1	1.00(0.08-12.10)	1.00	NA	3	0.88(0.31-2.51)	0.81	0	3	1.01(0.63-1.61)	0.97	0
	NS	1	1.07(0.02-51.87)	0.97	NA	1	1.47(0.11-19.96)	0.77	NA	NA	NA	NA	NA
**Tumor size**^a^													
	≤ 4cm	1	0.93(0.72-1.20)	0.56	NA	NA	NA	NA	NA	2	0.91(0.63-1.30)	0.59	0
	4-7cm	2	0.89(0.65-1.22)	0.48	0	3	1.56(0.69-3.54)	0.29	0	4	0.90(0.58-1.40)	0.65	0
	7-16cm	1	0.99(0.67-1.46)	0.95	NA	NA	NA	NA	NA	1	1.07(0.66-1.75)	0.77	NA
	Mixed	2	0.92(0.18-4.73)	0.92	0	2	0.90(0.19-4.21)	0.89	0	NA	NA	NA	NA
	NS	1	0.74(0.02-27.80)	0.87	NA	3	1.03(0.36-2.93)	0.96	0	1	0.89(0.02-33.55)	0.95	NA
**Follow-up time (m)**													
	≥50	1	1.00(0.08-12.10)	1.00	NA	2	0.97(0.24-3.92)	0.96	0	2	1.19(0.27-5.14)	0.82	0
	<50	3	0.89(0.20-3.94)	0.88	0	6	1.34(0.69-2.58)	0.39	0	3	0.99(0.62-1.59)	0.96	0

**OS** = overall survival; **RFS** = recurrence free survival; **CSS** = cancer specific survival; **HR** = hazard ratio; **NA** = not available; **NS** = not specified

a one included study (Srivastava 2018) reported three separate subgroup analyses for the same data set (≤ 4cm, 4-7cm and 7-16cm).

### Sensitivity analysis

Based on sensitivity analysis, 2-year CSS, 5-year CSS, 3-year recurrence rate and 5-year recurrence rate, HR of OS, CSS and RFS were all robust, with consistent findings.

### Publication Bias

Proof of publication bias was not found according to the HR of OS (Begg's test, P=1.000; Egger's test, P=0.969; Figure-S1A), RFS (Begg's test, P=0.711; Egger's test, P=0.165; Figure-S1B) and CSS (Begg's test, P=0.806; Egger's test, P=0.900; Figure-S1C).

**Figure S1:**
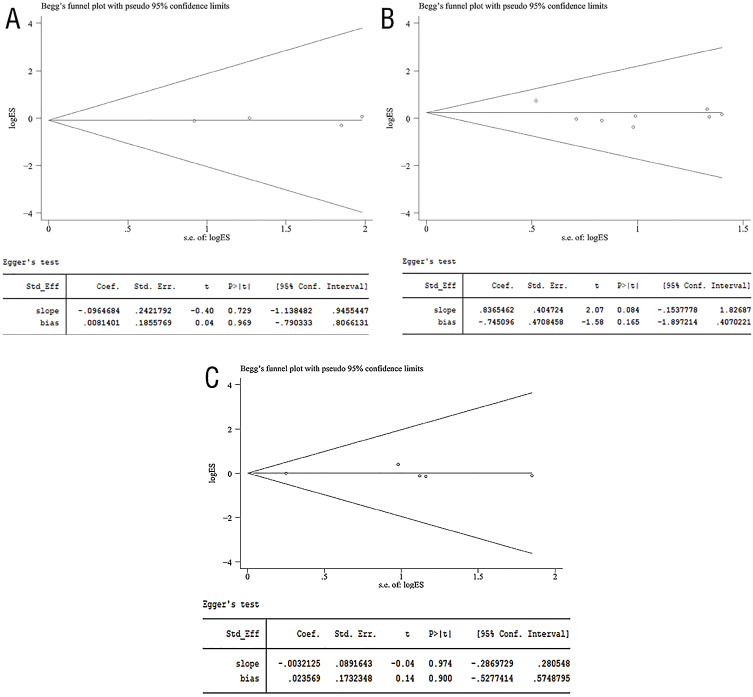
Begg's and Egger's tests for comparisons of HR of OS (a), RFS (b) and CSS (c) associated with PN versus RN.

## DISCUSSION

This is the first meta-analysis of the oncologic outcomes, surgical complications, perioperative outcomes and postoperative renal function between PN and RN for treating pT3a RCC. We found significantly better serum creatinine and eGFR levels in the postoperative period among patients undergoing PN compared with the RN group. PN offered equivalent oncologic outcomes among patients with pT3a RCC. Moreover, there were no significant differences between the two groups with regard to surgical complications, EBL and operative time.

The impact of oncologic outcomes was an indispensable factor when choosing PN or RN. Our meta-analysis found no significant differences regarding oncologic outcomes. There were also no differences in recurrence and metastasis. Andrade et al. (18) reported no differences in recurrence (2.9% vs. 1.4%, P=1.00) and metastasis (8.6% vs. 5.7%, P=0.74). Similarly, using the Cox proportional hazard model, Shvero et al. (20) demonstrated that surgical type was not a predictive factor for recurrence (P=0.978) and metastatic progression (P=0.972). Recently, some studies have demonstrated that PN offers equivalent cancer control compared with RN in treating large RCC, and Shvero et al. (23) suggested that PN yielded similar oncologic outcomes for pT3a RCC at the 5-year follow-up. Moreover, Thompson et al. (24) showed that compared with RN, PN had equivalent CSS and OS for masses between 4 and 7cm. In addition to these studies, two German centers reported that CSS was similar between two groups for tumors >7cm (25). Furthermore, the experience of successful PN even for pT3b renal tumors confined to the renal vein has also been published by some centers (26, 27). Although these studies from single or multiple centers support PN, we sought to analyze the data of surgical complications and postoperative renal function. Moreover, patients with robust renal function might be more suitable for RN because no survival advantage was found, though a significant positive margin difference favored RN (Figure-6C).

Surgical complications are a significant factor to consider when choosing PN or RN. We report that no significant differences were found regarding estimated blood loss (EBL). Our results also showed a trend toward a shorter operative time in the PN group (P=0.06), but without a significant difference, which was unlikely to be clinically significant. We observed a lack of a sufficient number of studies reporting surgical complications; indeed, only one of the included studies (Oh 2014) reported no significant differences in intraoperative complications (15.6% vs. 14.4%, P=0.842) and postoperative complications (13.3% vs. 12.4%, P=0.844) among pT3a RCC patients (14). However, EORTC 30904 found that PN was associated with more complications than RN, mostly hemorrhagic (28). In fact, the possible risk might be greater for more complicated and larger RCC, which requires a wider parenchyma resection, longer warm ischemia time and renal function reconstruction (29, 30). Therefore, our findings suggest that the potential advantages of PN need to offset the possibility of high surgical risk, especially for larger RCC.

The influence of kidney functional protection is essential when comparing PN and RN. Recently, some studies have demonstrated an association of RN with worse eGFR and a higher danger of cardiovascular events than PN (6, 31, 32). Furthermore, worse renal function has been associated with all-cause mortality and some cardiovascular risk factors, including increased inflammatory factors, anemia, artery calcification, endothelial dysfunction, left ventricular hypertrophy and high levels of apolipoprotein (33). A study including 1331 patients showed that the risk of cardiovascular events after nephrectomy was significant and that PN could independently reduce the risk of cardiovascular events compared with RN after interpreting latent confounders and selection biases secondary to baseline cardiovascular risk Kim et al. (34). Additionally, in a systematic review and meta-analysis of 34 included articles, Lane, et al. (35) found a cumulative 61% decrease in the risk of severe chronic kidney disease (CKD) and a 19% risk decrease of all-cause mortality for patients undergoing PN. Although EORTC 30904 suggested that the favorable effect of PN on postoperative eGFR did not lead to improved OS with a median follow-up of 9.3 years (3, 28), patients undergoing PN would undoubtedly have higher survival quality. These findings may be explained by recent studies favoring the concept that CKD is not equivalent (35). According to recently published studies, patients have a strong annual reduction in renal function with preexisting CKD (CKD-M) compared to surgical CKD (CKD-S), close to 5% versus 0.7%. Additionally, Lane et al. (36) suggested higher rates of progressive reduction in kidney function, all-cause mortality and non-renal cancer mortality for CKD-M compared to CKD-S, whereas CKD-S had better survival, with no CKD for a median follow-up of 9.4 years. Moreover, they confirmed the significance of renal functional protection by demonstrating an association between baseline eGFR of 45mL/min and worse results after surgery (36).

Some limitations should be considered in our meta-analysis. First, our results might have been influenced by potential bias because retrospective studies and conference abstracts were excluded. Second, some included studies did not completely match some important factors, such as tumor size, which may have an impact on final outcomes. Third, we were unable to completely control for confounding factors (for example, surgical approach), which were unavailable in some articles, that can influence the final results. Fourth, some data (3-year all-cause mortality, 5-year all-cause mortality, 3-year recurrence rate, 5-year recurrence rate, 2-year CSS and 5-year CSS) were extracted from survival curves, which may have led to deviations from the real data. Fifth, the limited number of studies regarding surgical complications and perioperative outcomes might have resulted in unreliable estimates. Sixth, there was significant heterogeneity (65%-81%) for some comparisons (3-year recurrence rate and 5-year recurrence rate), which would weaken the reliability of these results.

## CONCLUSIONS

Our meta-analysis suggests that PN may be more suitable for pT3a RCC, as it offers similar oncologic control and better renal functional preservation. Nevertheless, due to the inherent limitations of this meta-analysis, additional large-scale, high-quality articles are required to better determine the role of PN in complicated clinical situations.

## ABBREVIATIONS

RN: Radical Nephrectomy
PN: Partial Nephrectomy
RCC: Renal cell carcinoma
OS: Overall survival
CSS: Cancer-specific survival
RFS: Recurrence-free survival
EBL: Estimated blood loss
eGFR: Estimated glomerular filtration rate
RCT: Randomized control trial
pT3a: Pathological T3a
HR: Hazard ratio
NOS: Newcastle-Ottawa Scale
CIs: Confidence intervals
RR: Risk ratios
WMD: Weighted mean difference
